# Chemical Analysis of *Lepidium meyenii* (Maca) and Its Effects on Redox Status and on Reproductive Biology in Stallions †

**DOI:** 10.3390/molecules24101981

**Published:** 2019-05-23

**Authors:** Simona Tafuri, Natascia Cocchia, Domenico Carotenuto, Anastasia Vassetti, Alessia Staropoli, Vincenzo Mastellone, Vincenzo Peretti, Francesca Ciotola, Sara Albarella, Chiara Del Prete, Veronica Palumbo, Luigi Esposito, Francesco Vinale, Francesca Ciani

**Affiliations:** 1 Department of Veterinary Medicine and Animal Production, University of Naples Federico II, 80137 Naples, Italy; stafuri@unina.it (S.T.); vincenzo.mastellone@unina.it (V.M.); vincenzo.peretti@unina.it (V.P.); francesca.ciotola@unina.it (F.C.); sara.albarella@unina.it (S.A.); chiara.delprete@unina.it (C.D.P.); veronica.palumbo@unina.it (V.P.); luigespo@unina.it (L.E.); ciani@unina.it (F.C.); 2 UNMSM, Universidad Nacional Mayor San Marcos, Lima 11-0058, Peru; domenicar@alice.it; 3 Institute for Sustainable Plant Protection, National Research Council, 80055 Portici (Na), Italy; anastasia.vassetti@ipsp.cnr.it (A.V.); alessia.staropoli@unina.it (A.S.); francesco.vinale@ipsp.cnr.it (F.V.); 4 Department of Agricultural Sciences, University of Naples Federico II, 80055 Naples, Italy

**Keywords:** *Lepidium meyenii* (Maca), stallion semen, oxidative stress, antioxidants, chemical analysis

## Abstract

The present study was conducted to assess the chemical composition of Yellow Maca (*Lepidium meyenii*) and its biological activity on stallions following oral administration of hypocotyl powder. Maca was subjected to methanolic extraction and the chemical analysis was carried out by LC-MS-QTOF (liquid chromatography-mass spectrometry). Our results showed that Maca contains some effective antioxidants, a high percentage of glucosinolates, and other important components with a high antioxidant capacity. To evaluate the plant biological activity in stallions fed with Maca powder for 60 days, the redox status and some reproductive parameters were investigated. Blood and semen samples were collected at 0, 30, 60, and 90 days from the beginning of this study. Blood samples showed a decrease of the reactive oxygen metabolites, evaluated by d-ROMs test, and an increase of the antioxidant barrier in terms of biological antioxidant potential (BAP test), powerful oxidant capacity (OXY-Adsorbent test), and thiols evaluation (-SHp test). Furthermore, semen samples showed a positive trend during Maca administration in the following parameters: ejaculate volumes and sperm concentrations, total and progressive motility, and acrosome integrity.

## 1. Introduction

Maca (*Lepidium meyenii* Walp), belonging to the Brassicaceae family, was discovered more than 2000 years ago in the Andes highlands of Peru, where it grows exclusively between 3500 and 4500 m above sea level [1]. The hypocotyls have been widely used as a nutritional supplement and in folk medicine to increase fertility and sexual function [2]. The dried hypocotyls of Maca are rich in high nutritional value elements, such as carbohydrates, proteins, lipids, essential amino acids, and free fatty acids. Furthermore, Maca contains several secondary metabolites, such as macamides, macaridine, alkaloids, and glucosinolates [3]. The most abundant glucosinolates detected in Maca are aromatic glucosinolates, namely benzyl glucosinolate(glucotropaeolin) and *m*-methoxybenzyl glucosinolate (glucolimnanthin). Maca has been mainly classified in three ecotypes according to the color of the hypocotyls: red, yellow, and black. These showed different biological activity depending on the type of cultivation, processing, and extraction, and on the concentration of different bioactive metabolites [3,4]. The main chemical components of Maca were considered to be responsible, among other things, for its aphrodisiac, immunostimulant, and energizing properties [5,6].

Previous studies have demonstrated the effects of Maca on spermatogenesis, sperm count, and sperm motility [1,7]. Furthermore, in a rat model Maca restored the spermatogenesis decrease when altitude increases [8]. Additionally, diet supplementation of Maca showed a beneficial effect on semen quality of hypofertile and fertile stallions [9], as well as on the quantity of fresh semen and the quality of semen after storage at 5 °C up to 72 h [10].

The use of artificial insemination (AI) has greatly increased in recent decades. AI has several advantages compared to natural service, including higher pregnancy rates and reduced transportation costs of a mare to the stallion, with, consequently, lower stress and prevention of a stallion overuse. Semen used for AI should be preserved from damage occurring due to oxidative stress [10]. A strategy to prevent oxidative damage to spermatozoa may be found in food supplementation with antioxidants. The presence of compounds, such as macamides and glucosinolates, which are hydrolisated to isothiocyanates by myrosinase, allows Maca to scavenge free radicals and protect cells from oxidative stress (OS). Reactive oxygen species (ROS) are produced in different sites in mammalian bodies [11], and were found to accumulate during the normal testicular spermatogenesis and steroidogenesis [12]. Physiological low levels of ROS play an important role in processes such as capacitation, hyperactivation, acrosome reaction, and sperm-oocyte fusion, thus ensuring appropriate fertilization, whereas high levels of ROS may determine sperm pathologies, such as ATP depletion and loss of sperm motility and viability [13]. ROS activity is of major concern for sperm quality both in vivo and during in vitro incubation, as well as during its cool storage [14]. The cellular antioxidant defense systems are able to control the deleterious effects of ROS, but if an imbalance between oxidant and antioxidant systems determines ROS accumulation, OS may occur [15,16,17].

In the present study the chemical composition of Yellow Maca (*Lepidium meyenii*) was investigated by using LC-MS- QTOF. Moreover, the biological effects on stallions orally fed during the breeding season with Maca hypocotyl powder was evaluated in terms of antioxidant activity and semen quantity and quality.

## 2. Materials and Methods

### 2.1. Plant Material

**Figure 1:**
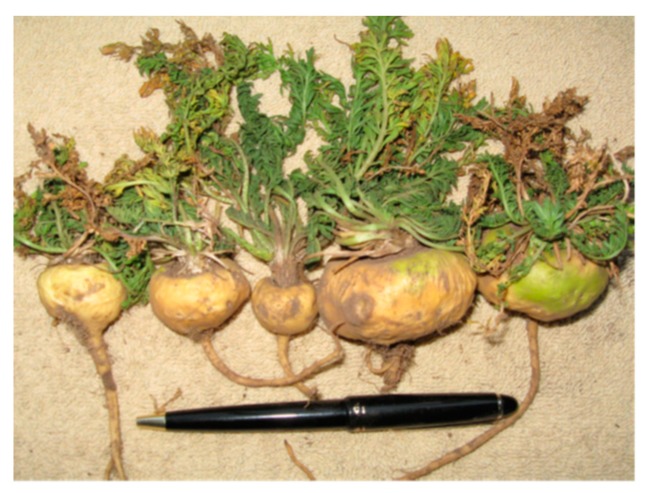
Typical roots of Yellow Maca from Junin district, Peru.

Yellow Maca hypocotyls used in this study were harvested in the district of Junin, in the Andean Highlands of Peru (4100 m above the sea level). The taxonomic identification of the plant was performed by Professor Domenico Carotenuto (Figure 1).

Maca powder was obtained from hypocotyls exposed for two months at extreme temperature cycles, strong light conditions, and atmospheric pressure typical of the high altitude environment, using the traditional open-field drying process. After drying, hypocotiles were selected, washed, ground to obtain a flour with a particle size of 0.8 mm, and stored until used.

### 2.2. Extraction and Chemical Analysis of Maca

A total of 3 g of dried sample was extracted with 30 mL of methanol (MeOH) for 30 min under ultrasonic treatment and centrifuged. The solution was stored at 4 °C and filtered through a 0.22 µm membrane syringe filter before injection into the LC system. All analyses were performed on an Agilent high performance liquid chromatograph (HPLC) 1260 Infinity Series (Agilent Technologies, Santa Clara, CA, USA) equipped with a DAD (Diode Array Detector) system (Agilent Technologies) and coupled to a quadrupole-time of flight (Q-TOF) mass spectrometer model G6540B (Agilent Technologies) with a Dual ESI source (Agilent Technologies). Separations were performed on a Zorbax Eclipse Plus C-18 column (4.6 × 100 mm, with 3.5 µm particles, Agilent Technologies), held at a constant temperature of 30 °C. For elution, mobile phase consisted of A: 0.1% (*v/v*) formic acid (FA) in water (H_2_O) and B: 0.1% formic acid (FA) in acetonitrile (ACN). Gradient elution was done at a flow rate of 0.3 mL/min, as follows: 0–1% B at 0–5 min, 1–30% B at 5–20 min, 30–50% B at 20–30 min, 50–70% B at 30–40 min, 70–95% B at 40–60 min, 95% B at 60–75 min, and 95–100% B at 75–80 min (as equilibration time). The injection volume was 5 µL. UV spectra were collected by DAD, setting the detection wavelength at 220 and 280 nm. MS and MS/MS parameters were set using the Agilent MassHunter Data Acquisition Software, rev. B.05.01 (Agilent Technologies). The system operated in positive and negative ion mode and for both cases MS spectra were recorded in *m/z* 50–1000 range as centroid spectra, with a speed of 1.5 spectra/s. The capillary was maintained at 4000 V, fragmentor voltage at 180 V, cone 1 (skimmer 1) at 45 V, Oct RFV at 750 V. Gas flow rate was set at 11 L/min, at 350 °C, and the nebulizer was set at 45 psig. A standard solution was infused by using an Isocratic pump (1260 Infinity Series, Agilent Technologies) in order to perform the real-time lock mass correction. The solution consisted of two reference mass compounds: purine (C_5_H_4_N_4_ at *m/z* 121.050873, 10 µmol/L) and hexakis (1H,1H, 3H-tetrafluoropentoxy)-phosphazene (C_18_H_18_O_6_N_3_P_3_F_24_ at *m/z* 922.009798, 2 µmol/L) [18,19,20]. Flow rate was set at 0.06 mL/min, while the detection window and the minimum height were set at 1000 ppm and 10,000 counts, respectively, for reference mass correction. MS/MS spectra were recorded in *m/z* 50–500 mass range, with a speed of 2 spectra/s. Collision energy was set at 20 eV.

A recovery study was done according to Zhou et al. [18]; recovery percentage was calculated for three standard compounds, namely benzyl glucosinolate, *m*-methoxybenzyl glucosinolate, and *N*-benzylhexadecanamide, resulting in 96.51%, 97.62%, and 98.15%, respectively. Raw data were evaluated using Mass Hunter Qualitative Analysis Software, rev B.06.00 (Agilent Technologies), and compound identification was carried out using an in-house database (containing about 2000 different plant metabolites), and by comparison with standard compounds or with data present in the literature [18,21,22,23]. Among the detected molecules, only those with a mass error below 10 ppm and a sufficient score (>80% similarity) were reported.

### 2.3. Animals and Experimental Design

In the present study, active breeding healthy stallions weighing 400 to 600 kg were included. The study was performed in accordance with the code of ethics (Legislative Decree 26–04/03/2014) and the protocol and procedures were approved by the Ethics Committee of Department of Veterinary Medicine and Animal Production at the University of Naples Federico II, Italy (Protocol Number 0003909).

The study was conducted during the breeding season (lasting from February to July) starting from the beginning of April to the end of July. Animals of both control (C; *n* = 5) and treated (M; *n* = 5) groups received an identical diet based on hay and concentrate pellets. In M the concentrate was supplemented daily with 20 g of Yellow Maca powder, resulting in an average dosage of 4 g Maca/100 kg body weight (minimum: 3 g Maca/100 kg; maximum: 5 g Maca/100 kg) for 60 days. Blood and semen specimens were collected at the beginning of the experiment (D0) and every month (D30, D60, and D90, respectively) for a total of 4 samplings for each stallion. The dosage was based on studies performed on humans [1,24] and animals [8,10,25,26]. Blood analysis was used to monitor oxidative stress. Semen was collected from stallions of treated group (M) and both quantitative (ejaculate volume, concentration, and total sperm count) and qualitative (total and progressive motility and acrosome integrity) determinations were performed on 4 animals according to previous studies carried out in our laboratory [10,27,28]. 

### 2.4. Systemic Oxidative Stress Status

All kits for the evaluation of OS status were purchased from Diacron International, Grosseto, Italy. The pro-oxidative status was evaluated by measuring hydroperoxides in the serum using the d-ROMs test; the measurements of antioxidant capacity in serum samples were performed by BAP-test, OXY-Adsorbent test, and thiols by -SHp test. The ratio between the values of d-ROMs and OXY (×100) (Oxidative Stress index—OSi) is an arbitrary value, used as an index of plasma redox status; high values indicate a higher concentration of oxidized molecules than non-enzymatic antioxidants [29]. 

The d-ROMs test is a photometric test that allows the oxidant capacity of plasma to be determined, mainly related to the level of hydroperoxides (a subclass of reactive oxygen metabolites, ROM); it measures the oxidant ability of a serum sample towards a particular substance used as an indicator (chromogen, e.g., *N,N*-diethylparaphenylendiamin), producing a pink-colored derivative that is photometrically quantified at 505 nm [30]. The intensity of the developed color is directly proportional to the concentration of ROMs, according to the Lambert-Beer’s law, and is expressed as Carratelli Units (1 CARR U = 0.08 mg hydrogen peroxide/dl).

In the Biological Antioxidant Potential (BAP) test the antioxidant power of the serum is evaluated by measuring the capacity of the sample to reduce a solution of ferric chloride from Fe^3+^ to Fe^2+^. This test measures the directly-active fraction of the anti-oxidant barrier, encompassing both exogenous antioxidants (vitamin C, vitamin E, carotenoids, bioflavonoids) and endogenous ones (bilirubin, uric acid, cholesterol, protein). Some of these substances exhibit a scavenger-type activity, i.e., they neutralize free radicals by interacting directly with them. The values of the BAP tests are expressed as μM of a sample. 

The OXY-Adsorbent test assesses the ability of the plasma to counteract the oxidation capacity of a hypochlorous acid (HClO) solution, which is both a powerful and physiological oxidant and is able to mimic situations occurring in vivo. Bilirubin, uric acid, vitamins C and E, albumin, and in general, macromolecular complexes that act as a shock absorber against free radicals, such as glycoproteins, help curb the oxidizing action of HClO. The values of the OXY-Adsorbent tests are expressed as μmol HClO/L of a sample.

The -SHp test measures thiols, organic compounds having a sulfhydryl group (-SH), that represent a significant qualitative component of the plasma antioxidant barrier against free radicals. Thiols also include cysteine and lipoic acid.

### 2.5. Statistical Analysis

The results are expressed as mean ± standard deviation (SD). Levels of OS in blood samples in C and M groups were compared using the Student *t*-test, as the data were normally distributed. A *p* value less than 0.05 was considered to be statistically significant. Due to the reduced number of semen samples obtained exclusively from animals fed with Maca (M), the Student *t*-test was performed on quantitative parameters of stallion semen between the samples collected at D30, D60, and D90, while samples collected at D0 were considered as controls.

## 3. Results

### 3.1. Chemical Analysis of Maca

LC-MS analysis (Figures S1 and S2) allowed to detect 88 different compounds, of which 80 were detected in ESI positive ion mode and 8 in ESI negative ion mode. Raw data were compared both with the in-house database and with data present in the literature [18,21,22,23]. From this analysis, 13 compounds were identified in positive mode via database search and 5 compounds using literature data (Table 1). Only *N*-benzylhexadecanamide (Compound No. 16) was identified by comparison with a commercial standard (Table 1). In negative mode 2 compounds were identified by comparing data with both existing literature and standard compounds (benzyl glucosinolate and *m*-methoxybenzyl glucosinolate, Table 2). 

In Table 1 molecules belonging to alkaloids and alkamides are reported. Compounds 1,3-dibenzyl-2, 4, 5-trimethylimidazilium (called Lepidine B or Macaline B) belong to the former class (No. 1, 3–8 in Table 1). Alkamides are natural compounds found in many plants formed by fatty acids and different amide groups. Polyunsaturated fatty acids that are specific for Maca are called macaenes and the LC-MS analysis allowed one of them (5-oxo-6*E*, 8*E* octadecadienoic acid; N. 9 in Table 1) to be identified. Identification also allowed 9 macamides (No. 2, 10–18 in Table 1) to be found, which are amide derivatives of fatty acids exclusively found in Maca.

The metabolites reported in Table 2, benzyl glucosinolate and *m*-methoxybenzyl glucosinolate, are two important glucosinolates of Maca that may be converted into isothiocyanates when the plant is damaged [31,32].

### 3.2. Oxidative Stress Measurements in Blood Samples

The values of blood count did not show any difference between C and M groups, but they confirmed the good state of health of the animals during the whole experimental period (data not shown). Table 3 reports the pro- and antioxidant data of blood samples. The mean values obtained with the d-ROMs test showed a constant decrease in M compared to C group, with *p* < 0.1 at D60, the time point corresponding to the end of Maca administration (Table 3). Interestingly, the evaluation of the antioxidant barrier showed higher mean values of the OXY-Adsorbent, and BAP tests in M compared to C at D60 (*p* < 0.1) and -SHp at D90 (*p* < 0.1) (Table 3). A significant increase of BAP was highlighted at D90 (*p* < 0.05) in M versus C (Table 3).

OSi showed a progressive decrease in treated animals (M), as compared to controls (C), exhibiting the lowest value at D60, which corresponded to the end of Maca administration. Conversely, 30 days after suspension of supplementation (D90) a slight increase of OSi was observed; however, this value was lower than the correspondent in C and M at D0 (Table 4).

### 3.3. Semen Analysis

The parameters for semen quantity are shown in Table 5. The ejaculate volume increased gradually from D0 to D60 (*p* < 0.05), and decreased at D90. The sperm concentration and total sperm count steadily increased from D0 to D90 (*p* < 0.05), even if a slight decrease was observed at D60 (Table 5).

**Figure 2:**
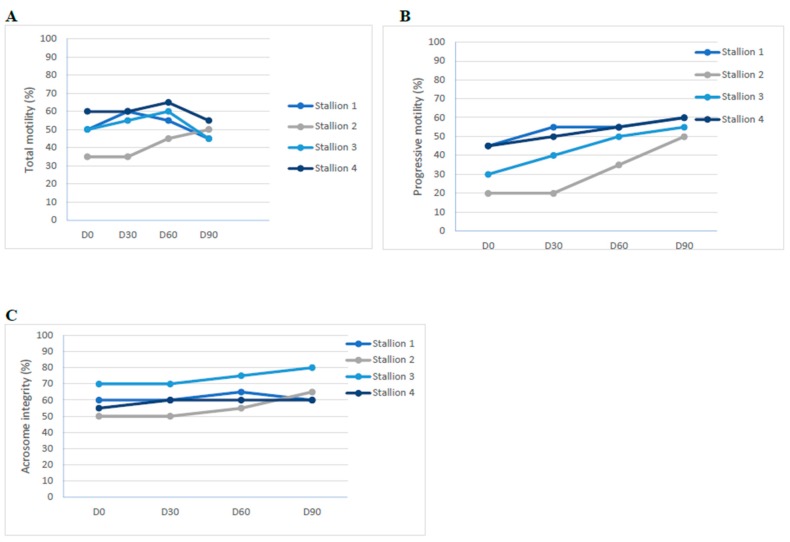
Trends of qualitative parameters of semen collected from four stallion fed with Maca during the experimental trial. (**A**) Total motility; (**B**) progressive motility; and (**C**) acrosome integrity.

The parameters for semen quality (total and progressive motility, and acrosome integrity) of 4 stallions in the M group are shown in Figure 2. The total motility reached a maximum value approximately after 60 days of Maca administration, while decreasing 30-days after Maca suspension (D90, Figure 2A). Conversely, both progressive motility and acrosome integrity increased gradually up to D90 (Figure 2B,C, respectively).

## 4. Discussion

In this study *Lepidium meyenii* (Maca) was characterized from the chemical point of view, and its main bioactive compounds were identified. Moreover, the antioxidant capacity of Maca diet supplementation was evaluated by redox status of blood, and by qualitative and quantitative semen parameters in stallions.

In our study yellow Maca hypocotyls were found to contain several compounds with antioxidant activity, such as alkaloids and glucosinolates; benzyl glucosinolate and *m*-methoxybenzyl glucosinolate are commonly the most abundant molecules found in fresh and dry hypocotils of Maca [31]. According to the analysis, the extract contains a percentage of 24.37% for benzyl glucosinolate and 4.31% for *m*-methoxybenzyl glucosinolate. The level of glucosinolates and their derivatives in the dry product is considered a good indicator of the quality of the product and of the effectiveness of the drying process [33]. Moreover, plant glucosinolates may be converted by myrosinase in isothiocyanates, bioactive metabolites with protective properties. Myrosinase is also present in the gut microflora of mammals [30,31]. 

Macamides are specific alkamides of Maca that are known for their antioxidant effect. Their concentrations are generally low, but their presence can be used to measure and standardize Maca quality [34,35]. The total amount of alkaloids in the extract is 40.74% and the percentage of other unidentified compounds is 31.21%. The drying process may affect the amount of macamides. Esparza et al. [33] showed that the traditional open-field drying method led the accumulation of macamide precursors. 

In the present study, the biological effect of Maca administration was evaluated by the systemic redox status. The antioxidant barrier evaluation provides indications on the type of defenses that the body can adopt to obtain the balance between pro- and antioxidant species. BAP, OXY-Adsorbent, and -SH tests measure the antioxidant capacity in blood from different points of view; thus, altogether give a complete picture of the antioxidant arrangement. In our study, a progressive increase of all antioxidants occurred during the first part of the experimental trial, with the maximum values obtained at D60, which corresponded to the suspension of diet supplementation with Maca. Subsequently, at D90 the values decreased, possibly as a consequence of the suspension of Maca supplementation, but they were significantly higher in M vs. C in BAP. Conversely, the detection of d-ROMs has shown a progressive decrease from D0 to D60 in M group, as compared to controls (C), while only at D90 an increase was highlighted. 

OSi allows a global view of the degree of oxidative stress, because it relates to the pro- and antioxidant status, thus avoiding data misinterpretation. The lowest value of OSi was found in M group at D60; this may indicate that after 60 days of Maca administration the redox balance in blood samples was hanging towards the antioxidant barrier. It is well known that high values of OSi could reflect an imbalance between oxidant and antioxidant systems, with an over-accumulation of ROS that can affect membrane structure and consequently changes in membrane fluidity [13,36]. However, despite the decrease of the antioxidant markers and increase of the oxidants at D90 with respect to D60, these final values were always higher in M group compared to the correspondent controls, and for the BAP were also statistically significant (*p* < 0.05 Student *t*-test). A range of the normal values of the redox status markers has been established for humans [30], but not for horses. In the literature very few data are available currently [37]; thus far, our results may provide knowledge for the study of the blood redox status in horses and could be useful in establishing species-specific references.

Finally, the biological effects of Maca administration as diet supplementation to stallions were evaluated in the reproductive season from April to July. Semen samples were collected at the same time points as blood samples (D0, D30, D60, D90). Quantitative semen parameters showed a progressive and significant increase during the experimental trial. The increase of ejaculate volume is in agreement with Gonzales et al. [1] and Melnikovova et al. [38], who highlighted this effect in rats and in humans, respectively. Sperm concentrations and total sperm count showed the same trend as the ejaculate volume, and this confirms the observations in a previous study by our group [10]. These results may also present interesting economic advantages, as an increased volume of the ejaculate allows preparation of a greater number of pellets to be used for AI and a greater concentration allows a higher probability of fertilization.

Data reported in Figure 2 show the trend of qualitative semen parameters of stallions belonging to M group. It was not possible to perform a reliable statistical analysis of the results, due to the reduced number of animals and for a lack of a control group, but the beneficial trend observed in terms of total and progressive motility and acrosomal integrity during the treatment with Maca confirms our previous findings [10].

In conclusion, this study revealed that Yellow Maca composition, and particularly the presence of specific bioactive metabolites, such as glucosinolates and macamides, may be responsible for the plant antioxidant activity. Moreover, our data showed that Maca supplementation positively affected the redox status and some reproductive parameters in stallions. 

## Figures and Tables

**Table 1:** Identification of the main *Lepidium meyenii* (Maca) metabolites by LC-MS-DAD and -qTOF analyses operating in positive ion mode (ESI +).

No.	Name	RT (min)	ESI(+)/Expected (*m/z*)	ESI(+)/Measured (*m/z*)	Delta (ppm)	Main Fragment (*m/z*)	λmax (nm)
1 *	(1*R*,3*S*)-1-methyltetrahydro-beta-5,6-hydride-carboline-3-carboxylic acid	13.842	233.1284	233.1276	3.431585341	91.054	215.5
2 *	*N*-(3-hydroxy-benzyl)-2*Z*-fivecarbon acrylamide	18.693	180.1019	180.1019	0.027	162.0914	220.1
3 *	1-dibenzyl-2-propane-4,5-dimethylimidazilium	18.997	227.1542	227.1546	−1.76091835	91.0544	219.5
4 **	3-benzyl-1,2-dihydro-*N*-hydroxypyridine-4-carbaldehyde	22.571	216.1175	216.1173	0.916936972	91.0543	213.2
5 *	1,3-dibenzyl-2,4,5-trimethylimidazilium	24.574	291.1854	291.1851	1.030271435	91.054	214.1
6 *	1,3-dibenzyl-2-phenyl-4,5-dimethylimidazilium	27.749	353.2014	353.2013	0.283124586	185.1072	221.1
7 **	*N*-ethyl-tetradecene ester	28.187	274.274	274.2743	−1.09379671	256.2637	221.2
8 **	1,3-dibenzyl-2-pentyl-4,5-dimethylimidazilium	29.578	347.2481	347.2482	−0.28797854	277.1705	222.7
9 **	5-oxo-6*E*,8*E*-octadecadienoic acid	40.444	295.2267	295.2266	0.338722751	277.2165	223.9
10 **	*N*-benzyl-5-oxo-6*E*,8*E*-octadecadienamide	43.789	384.2824	384.2822	0.520450585	306.2421	224.3
11 *	*N*-benzyl-9-oxo-12*E*-octadecenamide	44.976	386.2981	386.2979	0.517734879	261.2212	223.8
12 *	*N*-(3-methoxybenzyl)-(9*Z*,12*Z*,15*Z*)-octadecatrienamide	48.505	398.298	398.2976	1.004273182	381.2785	223.5
13 *	*N*-benzyl-(9*Z*,12*Z*,15*Z*)-octadecatrienamide	48.863	368.2875	368.288	−1.357635	351.2678	223.9
14 *	*N*-(3-Methoxybenzyl)-(9*Z*,12*Z*)-octadecadienamide	51.403	400.3137	400.3133	0.999216364	365.2838	224.3
15 *	*N*-Benzyl-(9*Z*,12*Z*)-octadecadienamide	51.868	370.3031	370.3034	−0.81014715	232.1694	224.7
16 *˟	*N*-benzylhexadecanamide	54.34	346.3031	346.3034	−0.86629314	221.2261	221.3
17 *	*N*-benzyl-9-Z-octadecenamide	55.169	372.3188	372.3194	−1.61152217	247.2423	225.9
18 *	*N*-benzyloctadecanamide	59.184	374.3345	374.3344	0.267140752	296.3172	225.5

Note: * compound identified by comparison with in-house database of plant secondary metabolites; ** compound identified by comparison with literature [18,21,22,23]; ^˟^ compound identified by comparison with a reference standard.

**Table 2:** Identification of the main *Lepidium meyenii* (Maca) metabolites by LC-MS-DAD and qTOF analyses operating in negative ion mode (ESI^−^).

No.	Name	RT (min)	ESI(-)/Expected (*m/z*)	ESI(-)/Measured (*m/z*)	Delta (ppm)	Main Fragment *(m/z)*	λmax (nm)
1 ^** ˟^	Benzyl glucosinolate	14.174	408.0423	408.0424	−0.24507263	274.9903 241.0027 166.0333	217.2
2 ^** ˟^	*m*-methoxybenzyl glucosinolate	15.421	438.0534	438.054	−1.36969602	358.0956 259.0126	219.4

Note: ** compound identified by comparison with literature [18,21,22,23]; ^˟^ compound identified by comparison with a reference standard.

**Table 3:** Mean values (±SD) of pro- and antioxidant data of blood samples calculated in C and M groups, evaluated during the experimental trial.

	d-ROMs (U CARR)		OXY-Ads (μmol HClO/mL)		BAP (μEq/L)		-SHp (μM/L)	
	**C**	**M**	**C**	**M**	**C**	**M**	**C**	**M**
**D0**	336	316.8	325	335.8	1759.3	1844.4	532.7	568.2
	(±34.4)	(±33.5)	(±9.9)	(±12.13)	(±58.8)	(±88.2)	(±57.5)	(±32.5)
**D30**	342.6	329.8	355.2	375.6	1652.4	1722.6	602.4	645
	(±37.3)	(±31)	(±26.3)	(±27.4)	(±122.7)	(±151.2)	(±70.0)	(±48.6)
**D60**	315.8	299.6	354	402	1746.4	1902.5	579.8	706.2
	(±12.1)	(±14.3)	(±58.5)	(±33.8)	(±193.2)	(±138.5)	(±67.7)	(±95.7)
**D90**	349.8	336.8	364.8	379.8	1580.8	1683 *	469.5	554.6
	(±28.7)	(±29.9)	(±43.2)	(±52.7)	(±51.6)	(±65.6)	(±49.6)	(±56.3)

C = control group; M = Maca treated group; D0, D30, D60, D90, samples taken at 0, 30, 60, 90 days from the beginning of the study, respectively; * *p* < 0.05 vs. corresponding C (Student *t*-test).

**Table 4:** OSi (%) is an arbitrary calculated value that indicates the plasma redox status for C and M groups.

OSi (%)		
	C	M
**D0**	103.4	94.3
**D30**	96.5	87.8
**D60**	89.2	74.5
**D90**	95.9	88.7

**Table 5:** Mean values (±SD) of some quantitative parameters of stallion semen fed with Maca, evaluated during the experimental trial.

	D0	D30	D60	D90
**Ejaculate volume (mL)**	38.38 ± 12.12	48.13 ± 17.50 *	60.88 ± 29.09 *	47.5 ± 23.63 *
**Sperm concentration (×10^6^/mL)**	124.38 ± 43.05	220.00 ± 90.69 *	178.88 ± 86.54 *	279 ± 126.87 *
**Total Sperm Count (×10^9^)**	3.68 ± 1.18	10.15 ± 6.22 *	8.20 ± 7.65 *	8.83 ± 4.36 *

Note: * *p* < 0.05 vs. D0 (Student *t*-test).

## Supplementary Materials

The following are available online, Figures S1 and S2.
