# Supplementary material for: Chemical Analysis of Lepidium meyenii (Maca) and Its Effects on Redox Status and on Reproductive Biology in Stallions
Source: Molecules. 2019 May 23;24(10):1981. doi: 10.3390/molecules24101981 (PMC6571579; doi:10.3390/molecules24101981)
Supplement: Supplementary file 1 [file molecules-24-01981-s001.pdf]

Supplementary Materials

Chemical analysis of *Lepidium meyenii* (Maca) and its effects on redox status and on reproductive biology in stallion

Simona Tafuri[1#], Natascia Cocchia[1#*], Anastasia Vassetti[3], Alessia Staropoli[3,4], Vincenzo Mastellone[1], Vincenzo Peretti[1], Francesca Ciotola[1], Sara Albarella[1], Chiara Del Prete[1], Veronica Palumbo1, Domenico Carotenuto[2], Luigi Esposito[1], Francesco Vinale[3] and Francesca Ciani[1]

[1] Department of Veterinary Medicine and Animal Production, University of Naples Federico II, Naples, Italy;
[2] UNMSM, Universidad Nacional Mayor San Marcos, Lima, Peru;
[3] Institute for Sustainable Plant Protection, National Research Council, Portici (Na), Italy;
[4] Department of Agricultural Sciences, University of Naples Federico II, Naples, Italy.
[#] The authors contributed equally to this work.
[*] Corresponding author: ncocchia@unina.it ; Tel: +39-81-2536017; Fax: +39-81-2536042; e-mail: ncocchia@unina.it

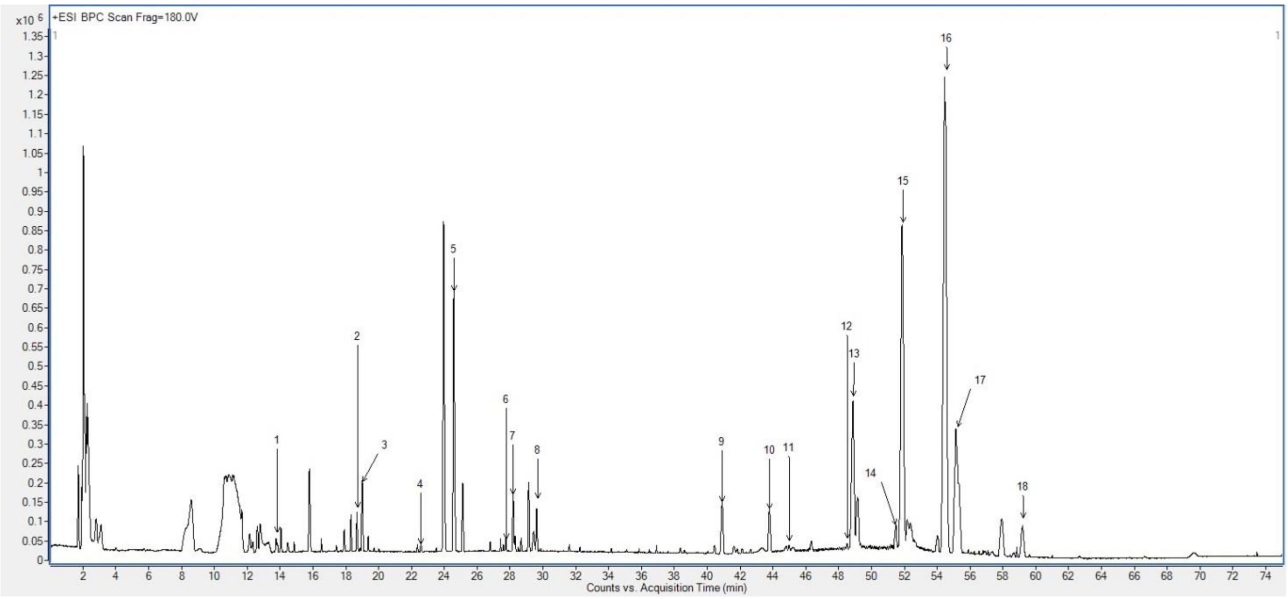

Figure 1. LC-MS chromatogram of MACA (*Lepidium meyenii)* extract obtained in ESI positive mode. Numbers correspond to the alkaloids identified as follows:**1**, (1*R*,3*S*)-1-methyltetrahydro-beta-5,6-hydride-carboline-3-carboxylic acid; **2**, *N*-(3-hydroxy-benzyl)-2*Z*-fivecarbon acrylamide; **3**, 1-dibenzyl-2-propane-4,5-dimethylimidazilium; **4**, 3-benzyl-1,2-dihydro-*N*-hydroxypyridine-4-carbaldehyde acid; **5**, 1,3-dibenzyl-2,4,5-trimethylimidazilium; **6**, 1,3-dibenzyl-2-phenyl-4,5-dimethylimidazilium; **7**, *N*-ethyl-tetradecene ester; **8**, 1,3-dibenzyl-2-pentyl-4,5-dimethylimidazilium; **9**, 5-oxo-6*E*,8*E*-octadecadienoic acid; **10**, *N*-benzyl-5-oxo-6*E*,8*E*-octadecadienamide; **11**, *N*-benzyl-9-oxo-12*E*-octadecenamide; **12**, *N*-(3-methoxybenzyl)-(9*Z*,12*Z*,15*Z*)-octadecatrienamide; **13**, *N*-benzyl-(9*Z*,12*Z*,15*Z*)-octadecatrienamide; **14**, *N*-(3-Methoxybenzyl)-(9*Z*,12*Z*)-Octadecadienamide; **15**, *N*-Benzyl-(9*Z*-12*Z*)-octadecadienamide; **16**, *N*-benzylhexadecanamide; **17**, *N*-benzyl-9-*Z*-octadecenamide; **18**, *N*-benzyloctadecanamide.

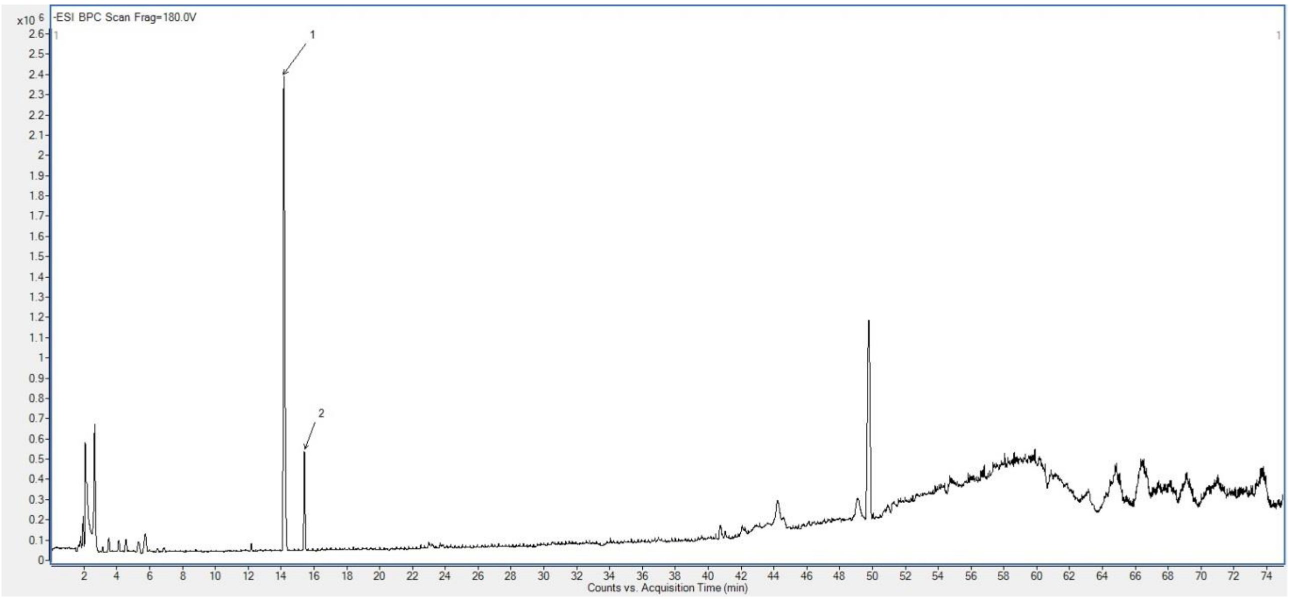

Figure S2: LC-MS chromatogram of MACA (*Lepidium meyenii)* extract obtained in ESI negative mode. Numbers correspond to the glucosinolate identified as follows: **1**,1-S-[(1E)-2-Phenyl-N-(sulfooxy)ethanimidoyl]-1-thio-β-D-glucopyranose; **2**, 1-S-[(1Z)-2-(3-Methoxyphenyl)-N-(sulfooxy)ethanimidoyl]-1-thio-β-D-glucopyranose
